# Near-circumferential Lower Body Lift: A Review of 40 Outpatient Procedures

**DOI:** 10.1097/GOX.0000000000002548

**Published:** 2019-12-30

**Authors:** Eric Swanson

**Affiliations:** From the Swanson Center, Leawood, Kans.

## Abstract

Lower body lift surgery has increased in popularity. A circumferential body lift or belt lipectomy is often recommended to treat skin redundancy. A drawback for this procedure is the midline scar bridging the lower back causing elongation of the gluteal cleft. Autoaugmentation methods have not been shown to provide a net increase in buttock volume.

**Methods:**

A retrospective study was undertaken among 40 consecutive women and men undergoing near-circumferential outer thigh and buttock lifts, including 21 lower body lifts (with abdominoplasty). All procedures were performed by the author as outpatients, under total intravenous anesthesia, without muscle relaxation and without prone positioning. Most patients (80%) had liposuction. Fourteen patients had simultaneous inner thigh lifts. Buttock fat transfer was used in 13 patients. Most patients had simultaneous cosmetic procedures of the face or breasts.

**Results:**

Fourteen patients (35%) experienced complications. One patient developed a deep venous thrombosis, detected by routine ultrasound screening on the day after surgery. Local complications included 3 patients with seromas (8%), 2 wound dehiscences (5%), and 1 infection (3%). Three patients (8%) returned for secondary outer thigh lifts. There were no complications related to fat injections.

**Conclusions:**

The near-circumferential lower body lift may be performed in healthy outpatients with attention to safe anesthesia, normothermia, limited blood loss, and operating times <6 hours. A scar across the posterior midline may be avoided. Fat injection safely restores gluteal volume. Secondary surgery may be recommended to treat persistent skin laxity.

## INTRODUCTION

Thighplasty is becoming more popular as patients lose large amounts of weight from dieting and after bariatric surgery. This surgery may also be indicated in patients who have developed skin laxity from aging.

The outer thighs may be lifted with the buttocks in an operation called an outer thigh lift, or outer thigh/buttock lift. This procedure may be done at the same time as an abdominoplasty. The combined procedure—outer thigh/buttock lift and abdominoplasty—is labeled a lower body lift. When the incision crosses the midline of the back, the term circumferential body lift or belt lipectomy is used.

Some surgeons believe that a circumferential incision is needed to obtain optimal results.^1,2^ However, in many patients, it is unnecessary to connect the incision across the back, particularly if there is no skin excess directly in the midline. Preservation of a skin bridge minimizes the risk of elongation of the gluteal cleft^3^ (plumber’s crack deformity) and avoids placing an incision directly over a pressure point.^4^

## PATIENTS AND METHODS

The study group included all patients treated by the author with near-circumferential outer thigh lifts from 2013 to 2019. No patients were treated with circumferential lifts during the study period. All patients underwent surgery at a state-licensed ambulatory surgery center. This retrospective study was determined to be exempt from Institutional Review Board oversight by Advarra Institutional Review Board (Columbia, MD).

### Preoperative Considerations

Preoperative antibiotics were given in the form of cefazolin (Ancef; GlaxoSmithKline, London, U.K.) 1 g, or clindamycin 600 mg (Cleocin; Pfizer, New York, NY) intravenously in patients who were allergic to cephalosporins. Oral antibiotics were typically continued in the form of Augmentin 500 mg twice daily for 5 days, or Cipro 500 mg twice daily in patients who were allergic to penicillin. Patients were instructed to abstain from smoking during the perioperative period, at least 2 weeks before and 2 weeks after surgery.

As part of venous thromboembolism prevention, patients underwent Doppler ultrasound scans of the deep veins of the lower extremities before surgery, on the day after surgery, and approximately 1 week after surgery.^5^ In patients undergoing liposuction of the abdomen or abdominoplasty, the abdomen was also scanned preoperatively to detect any possible hernias. No chemoprophylaxis was used. Sequential compression devices were used in patients undergoing surgery between 2013 and 2016.^5^

All patients were American Society of Anesthesiologists Class I or II. The maximum body mass index was <35 kg/m^2^. Simultaneous facial cosmetic surgery procedures, such as submental lipectomies or facial fat injections, typically required <30 minutes of additional operating time.

### Preoperative Marking

**Fig. 1.**
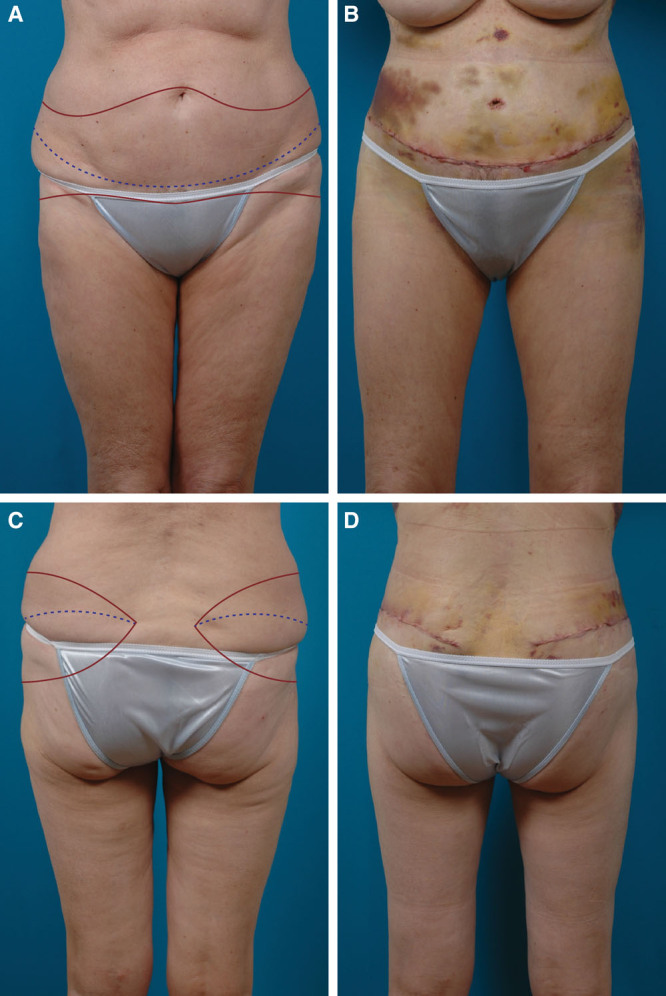
A 66-year-old woman with no history of massive weight loss is shown before (A, C) and 8 days after (B, D) a near-circumferential lower body lift with liposuction of the abdomen and flanks, and buttock fat injection. The planned incisions (red line) and level of the scar (hatched blue line) are indicated on the preoperative photographs (A, C).

Patients were marked in a standing position immediately before surgery (See Video 1 [online], which demonstrates preoperative marking, local anesthesia, surgery, fat injection, and postoperative photographs in a 40-year-old woman treated with an outer thigh lift) (See Video 2 [online], which displays a 50-year-old woman who underwent a lower body lift with simultaneous medial thigh lifts). The surgery was planned so that the scar would be concealed by panties and bikini bottoms. An elliptical resection pattern was drawn from the inguinal crease to a point lateral to the midline, below the dimple created by the posterior superior iliac spine. In patients undergoing a lower body lift, the marking was made in combination with the marking for the abdominoplasty (Fig. 1).

**Video 1.** This video demonstrates preoperative marking, local anesthesia, surgery, fat injection, and postoperative photographs in a 40-year-old woman treated with an outer thigh lift. From “Near Circumferential Lower Body Lift: A Review of 40 Outpatient Procedures”

**Video 2.** This video demonstrates a 50-year-old woman who underwent a lower body lift with simultaneous medial thigh lifts. From “Near Circumferential Lower Body Lift: A Review of 40 Outpatient Procedures”

Medial thigh lifts were also marked with the patient standing, so as to leave a linear scar along the inseam running from the adductor magnus origin to a point just distal to the area of skin laxity, usually at the level of the medial femoral condyle. The proximal dog ear was chased into the perineal crease as a J-extension.

### Anesthesia

A total intravenous anesthetic, consisting of a propofol infusion, was administered with a laryngeal mask airway and no muscle relaxation.^6^

### Surgery

Patients were prepped circumferentially in a standing position using chlorhexidine mixed with warm saline (Video 2). In patients undergoing simultaneous cosmetic breast surgery, the chest was prepped a second time and draped. The breast surgery was always performed first to optimize sterility. Prone positioning was never used.

The operating room temperature was maintained at 75°F. Body warmers (Bair Hugger; 3M Comp., Maplewood, MN), warm blankets, and warm infusion solutions were used to avoid hypothermia. Patient temperature was monitored with a temperature strip placed on the forehead.

In patients who underwent a simultaneous abdominoplasty, a 1-L solution of normal saline with 0.025% bupivacaine and 1:500,000 epinephrine was used to infuse the abdomen.^6^ The epigastrium and (frequently) pubic area were treated with liposuction. The lower abdomen was also treated in patients who elected to have simultaneous buttock fat transfer (Video 2). A traditional abdominoplasty was performed using scalpel dissection and removing the Scarpa fascia and fat from the lower abdomen.^7^ The rectus diastasis was repaired using 2 layers of running 0-Prolene (Ethicon, Inc., Somerville, NJ) sutures. The operating table was flexed to ensure low scar placement and avoid a vertical midline scar. A 3-layer closure was performed. The first layer approximated the Scarpa fascia using 2-0 Vicryl (Ethicon, Inc.) sutures. In the central third (pubic) portion of the wound, an extra “deep fascial anchoring” bite was taken in the rectus abdominis muscle fascia.^7^ The lateral wounds were left open, in continuity with the planned flank resections. A single drain was used, exiting the right pubic portion of the wound.

After the abdominoplasty, the patient was turned onto the left side. The right flank was infused with a 0.05% lidocaine and 1:500,000 epinephrine solution. Liposuction was performed. Additional local anesthetic (<50 mL of 0.5% lidocaine, 1:200,000 epinephrine) was injected. The outer thigh lift was performed, maintaining a layer of loose areolar tissue so as to preserve the lateral femoral cutaneous and iliohypogastric nerves, which course medial and inferior to the anterior superior iliac spine.^8^ The liposuction pretreatment facilitated the dissection. Scalpel dissection was used exclusively, reserving electrocautery for individual bleeders.

A 3-layer repair was performed using 2-0 Vicryl sutures for the Scarpa fascia, 3-0 Vicryl for the superficial fascia and dermis, followed by a 4-0 Monocryl (Ethicon, Inc.) running intradermal suture. The patient was turned onto the contralateral side, which was treated in the same manner. No undermining was performed, and no drains were used for the outer thigh lifts (a drain was used for the abdominoplasty).

The last step was subcutaneous buttock fat injection in patients who elected to have it. Gauze and Microfoam (3M Comp.) tape dressings were applied followed by a compression garment.

In patients who also underwent medial thigh lifts, the lower extremities were abducted slightly and externally rotated to provide exposure, with the patient positioned supine. The medial thighs were infused with a solution of 0.05% lidocaine and 1:500,000 epinephrine, similar to the flanks. Small volumes (eg, 30 mL per side) of 0.5% lidocaine with 1:200,000 were used to supplement this infusion.

In most cases, liposuction was done first. Scalpel dissection was used exclusively. No undermining of skin edges was performed. A 3-layer closure consisted of 2-0 Vicryl sutures to repair the subcutaneous fat layer, 3-0 Vicryl for dermis, and a 4-0 Monocryl intradermal suture. A noncircumferential gauze dressing was applied.

## RESULTS

**Fig. 2.**
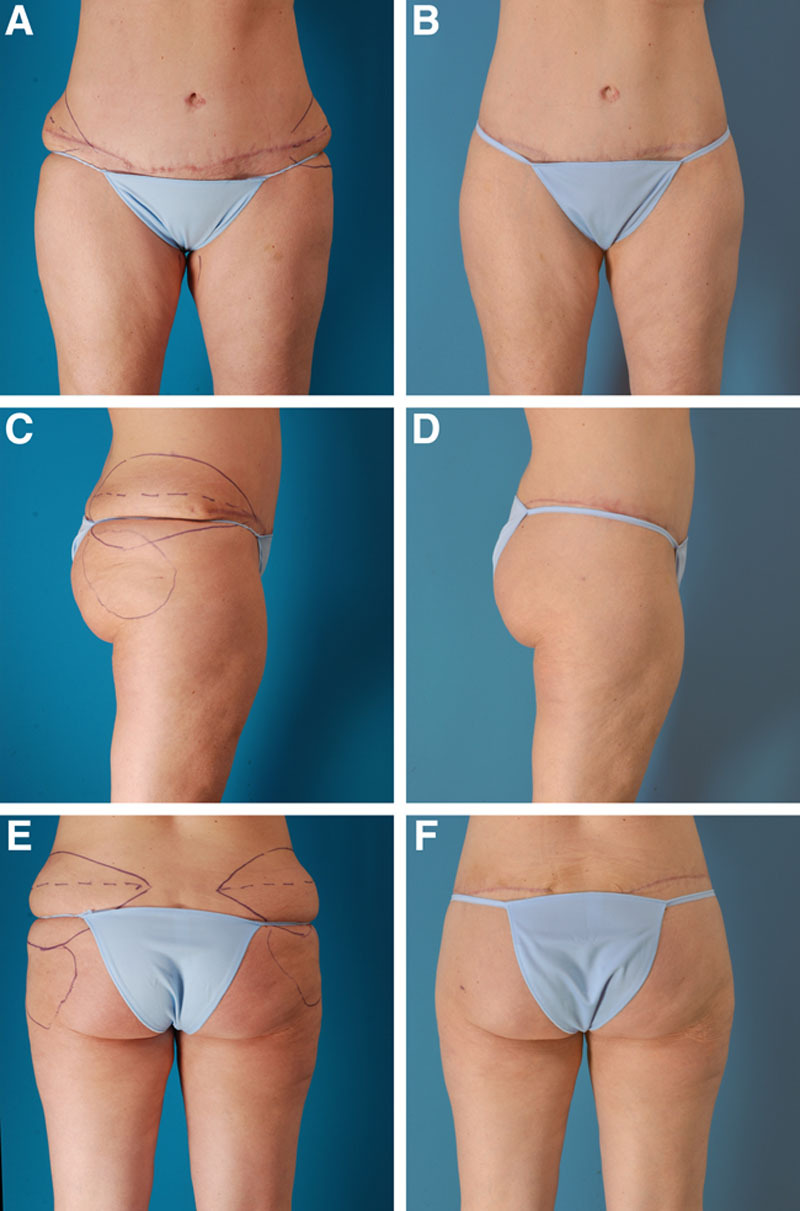
A 40-year-old woman with no history of massive weight loss is shown before (A, C, E) and 3 months after (B, D, F) outer thigh and buttock lifts, performed in combination with liposuction of the abdomen, flanks, arms, and axillae, and buttock fat injection. She had undergone a previous abdominoplasty and liposuction. This patient’s preoperative marking and surgery are provided as Video 1 [online] (which demonstrates preoperative marking, local anesthesia, surgery, fat injection, and postoperative photographs in a 40-year-old woman treated with an outer thigh lift).

**Fig. 3.**
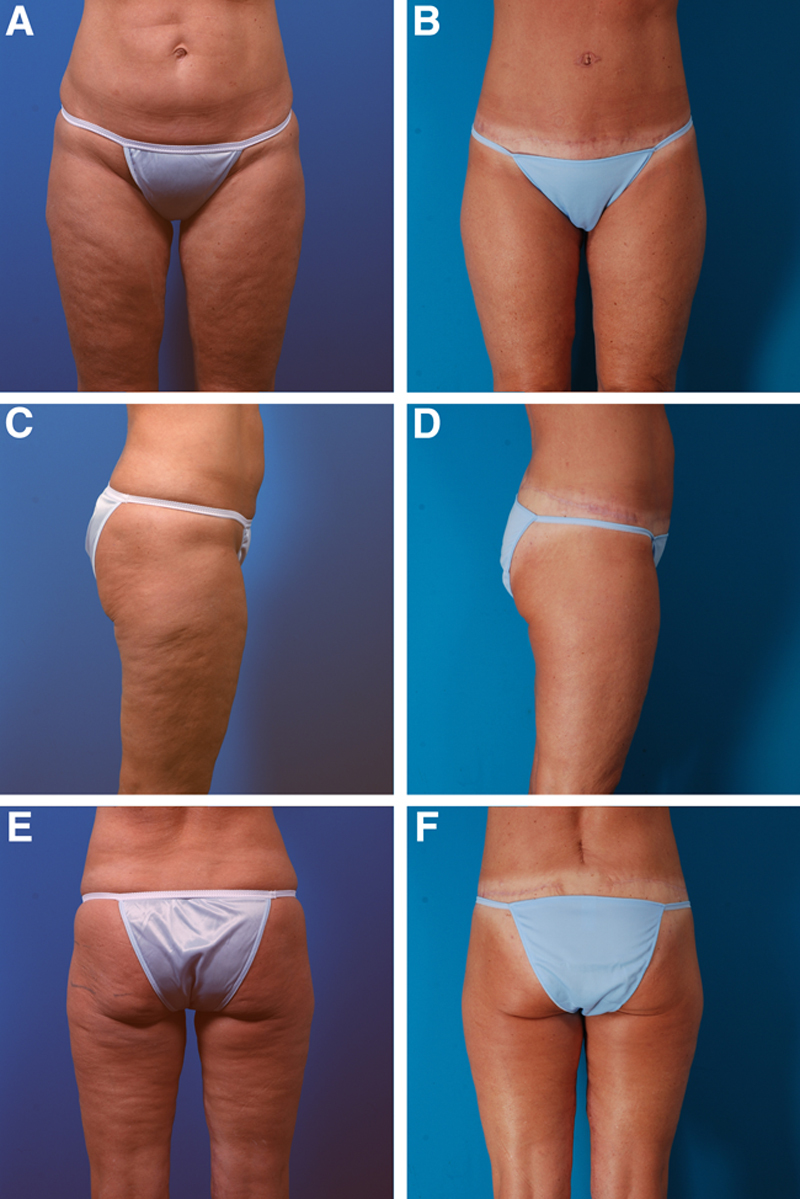
A 56-year-old woman with no history of major weight loss is shown before (A, C, E) and 16 months after (B, D, F) a lower body lift, liposuction of the abdomen and flanks, and secondary medial thigh lifts. Postoperatively, she has a vertical scar of the lower back from recent unrelated surgery on her lumbar spine (F). She had undergone previous lower body liposuction. She chose not to have buttock fat injection.

**Fig. 4.**
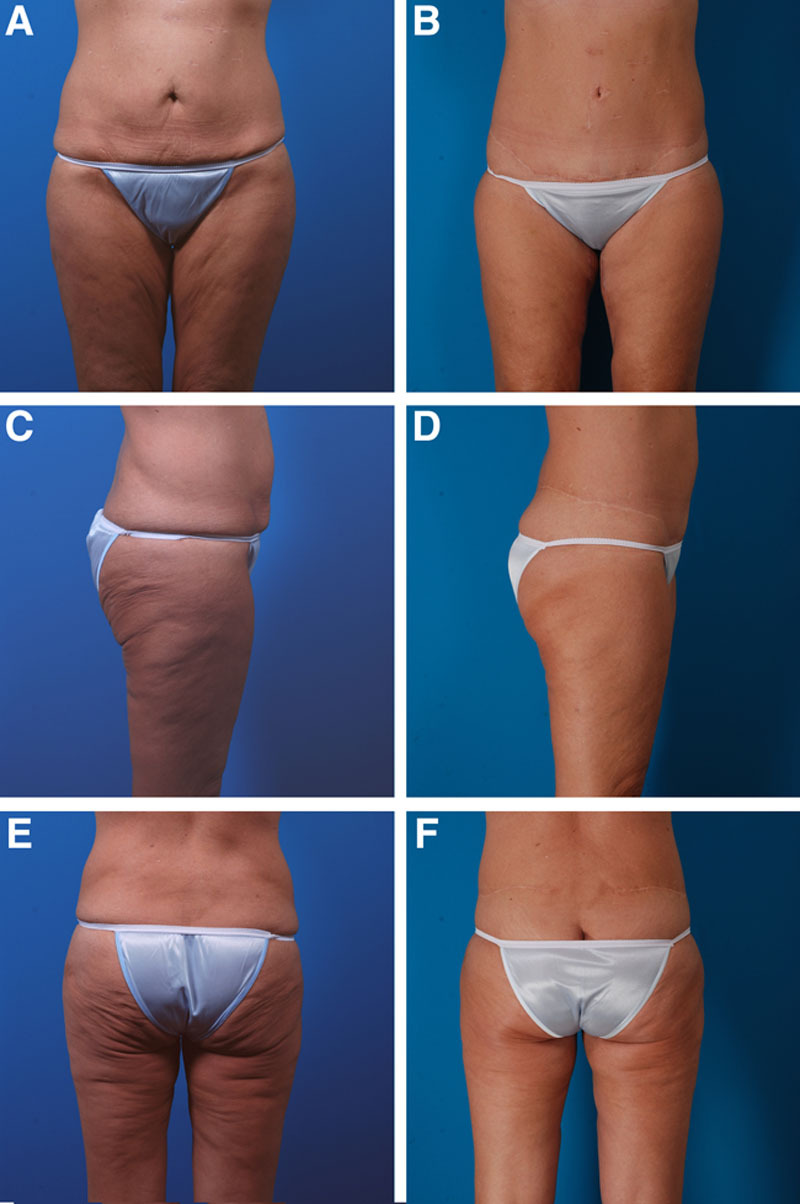
A 52-year-old woman had a previous bariatric operation, accounting for the scars from laparoscopies on her abdomen (A, C, E). She is seen 2 years after (B, D, F) a lower body lift; medial thigh lift; liposuction of the abdomen, flanks, outer thighs, and knees; and buttock fat transfer.

Forty men and women underwent near-circumferential outer thigh and buttock lifts (Table 1). This procedure was done without a simultaneous abdominoplasty in 19 patients (Fig. 2). Just over half of patients (53%) underwent lower body lifts, combining abdominoplasty with the outer thigh/buttock lifts (Figs. 3 and 4). Most patients (80%) were treated with liposuction (Table 2). Twenty-seven patients had simultaneous facial cosmetic surgery and 16 women had simultaneous cosmetic breast surgery (eg, breast augmentation and/or mastopexy). Fourteen patients (35%) had simultaneous inner thigh lifts (Figs. 3 and 4). Thirteen patients (33%) underwent buttock fat injection (Figs. 2 and 4).

**Table 1.** Data for 40 Patients Undergoing Outer Thigh/Buttock Lifts

	Value (%)
No.	40
Age, y	
Mean	46.7
SD	12.1
Range	22.5–75.6
Sex	
Woman	35 (88)
Man	5 (12)
Follow-up time, mo	
Mean	12.9
SD	17.2
Range	1–76
Body mass index, kg/m^2^	
Mean	25.2
SD	3.9
Range	18.9–34.4
Smoking status	
Nonsmoker	36 (90)
Smoker	4 (10)
Operating time, min*	
Mean	226
SD	83
Range	86–360
Right buttock fat volume (cc)	
Mean	232
SD	114
Range	70–445
Left buttock fat volume (cc)	
Mean	232
SD	114
Range	70–445

*Time includes simultaneous procedures on the face and breasts.

**Table 2.** Procedures Performed Simultaneously with Outer Thigh/Buttock Lifts in 40 Patients

*Procedure*	No. (%)
Liposuction	32 (80)
Facial cosmetic surgery	27 (68)
Abdominoplasty (lower body lift)	21 (53)
Cosmetic breast surgery	16 (40)
Inner thigh lifts	14 (35)
Fat transfer to buttocks	13 (33)

There were no cases of gluteal cleft elongation. Fourteen patients (35%) experienced complications (Table 3). One patient developed an asymptomatic deep venous thrombosis, detected by routine ultrasound screening on the day after surgery. This patient was referred to a local hospital where a filter was placed in her inferior vena cava as an outpatient the same day. The radiologist diagnosed May–Thurner syndrome, a congenital vascular anomaly causing compression of the left common iliac vein. This patient was prescribed rivaroxaban, 15 mg by mouth twice daily after insertion of the filter. The thrombus disappeared by the fourth postoperative day, as determined from a subsequent ultrasound scan, and she made an uneventful recovery. Three patients (8%) required aspirations in the office for seromas. Two patients had wound dehiscences that were repaired under local anesthesia in the office. A left flank wound dehiscence occurred in a woman who was in a motor vehicle accident 13 days after a lower body lift and had to climb out of a ditch. There were no complications related to fat injections. Three patients (8%) returned for a secondary outer thigh lift to treat persistent skin laxity.

**Table 3.** Complications and Reoperations in 40 Patients Undergoing Outer Thigh/Buttock Lifts

	No. (%)
No.	40
Complications	
No	26 (65)
Yes	14 (35)*
Scar deformity (dog ears and umbilical scar deformity)	5 (13)
Persistent skin laxity	3 (8)
Seroma	3 (8)
Wound dehiscence†	2 (5)
Cellulitis	1 (3)
Deep venous thrombosis	1 (3)
Surgical treatment of complications	
No	29 (72)
Yes	11 (28)
Reoperations (total IV anesthesia)	
Dog ear revision, umbilical scar revision‡	4 (10)
Revision for persistent skin laxity	3 (8)
Revision (local anesthesia)	
Revision of dog ears	2 (5)
Repair of wound dehiscence	2 (5)

*One patient had both a seroma and a wound dehiscence.

†One patient sustained a dehiscence in a motor vehicle accident.

‡Performed at the time of a subsequent unrelated operation under total IV anesthesia.

IV, intravenous.

## DISCUSSION

Afrooz et al^3^ report that 80% of patients develop either moderate or severe gluteal cleft elongation after circumferential lower body lifts. According to these authors, this deformity occurs because of direct elevation of the gluteal cleft, and medial redistribution of excess inferior gluteal tissue into the cleft.^3^ The elongated cleft may require excision and direct closure, leaving a vertical scar.^3^ As an alternative to a lower body lift, Hurwitz et al^9^ describe an oblique flankplasty in combination with lipoabdominoplasty, avoiding a circumferential scar. The flank scars course obliquely above the bikini line, ending just below the bra line posteriorly.

Hamra and Small^10^ used the term “cosmetic body lift” to describe a 270-degree extended lipoabdominoplasty. This operation modified the extended abdominoplasty described by Hunstad and Repta,^11^ who carried the lateral incision posteriorly, but without turning the patient either on the side or prone. The near-circumferential lift provides an almost complete circumferential lift, but stops short of 360 degrees. The scar is located so as to avoid any encroachment on the buttock itself. A slight curve is aesthetically preferable to a straight line.^10–12^ Preservation of a skin bridge avoids a continuous scar across the back, which can bridge this cosmetically sensitive area, interfering with the natural “small of the back” depression. The rationale is similar to mastopexies, in which the surgeon avoids a continuous scar across the lower sternum. Although some patients with an extreme deformity after massive weight loss may require a 360-degree resection, the author has not found a continuous incision necessary since adopting the near-circumferential method.

Supine and lateral positioning^2,6,10,12,13^ is preferred by the author over prone/supine positioning. A disadvantage of prone positioning for combined body/breast procedures is that the breast surgery must be done after prone positioning for body contouring surgery so as to avoid pressure on the breasts. Patient must be repositioned supine. By this time, sterility may be compromised. Some operators reprep and redrape the patient, change gowns and gloves, and open a new instrument set,^14^ but these steps lengthen the operating time.

Adequate regional anesthesia using superwet infusions reduces the need for central “masking” anesthesia.^6^ The patient ventilates spontaneously. The goal is to disturb the patient’s physiology as little as possible during surgery. Mechanical ventilation can cause respiratory alkalosis and secondary hypokalemia.^6^ “SAFE” (Spontaneous breathing, Avoid gas, Face up, Extremities mobile)^15^ anesthesia reduces recovery room time and almost eliminates nausea and vomiting.^6^

Surgeons should be experienced in the individual procedures and able to do them expeditiously to avoid protracted operating times. The operating time for a lower body lift (outer thigh lifts + abdominoplasty) is typically about 3 hours. Medial thigh lifts require approximately 1 hour. The author does not plan operations that are expected to exceed 6 hours. Consequently, a urinary catheter is not used routinely during surgery. Usually, the patient voids on her own or is catheterized in the recovery room.

Massive weight loss patients have an increased risk of impaired wound healing and an increased prevalence of medical comorbidities including diabetes and hypertension.^1,16^ Preoperative optimization of the patient’s nutritional status, including iron, calcium, and vitamin B_12_ levels is recommended.^16^ Low albumin levels, iron deficiency, and vitamin A, D, E, and K deficiency are common.^17^ Postbariatric patients, particularly those who have had malabsorbtive procedures (eg, gastric bypass and duodenal switch), are often anemic.^1^ Preoperative iron supplementation is frequently recommended.^1^

### Gluteal Augmentation

Flap transposition has been used in an effort to restore buttock volume. A variety of methods have been published.^18–23^ A medially based deepithelialized flap may be dissected from the flank and tunneled over the gluteus maximus.^18,22^ Bertheuil et al^23^ describe a lipo-body lift procedure, which lifts the tissues superomedially without undermining and reportedly improves buttock projection.^23,24^ However, measurements on matched photographs fail to show a benefit.^4^ Moreover, 40% of patients develop a wound dehiscence.^23^ Sozer et al^21^ describe a split gluteal muscle flap, flipping the gluteus maximus muscle 180 degrees. However, lateral photographs do not confirm increased gluteal projection.^4^

Hunstad and Repta^25^ recommend a purse-string autoaugmentation. Intraoperative photographs show a pleasing increase in projection. Whether there is a lasting benefit is unclear. A problem for any autoaugmentation method, whether in the breast or buttock, is the lack of a net increase in tissue.^4^ Photographs of long-term results are lacking. Srivastava et al^22^ report a significantly higher complication rate (42.5%), mostly wound dehiscences, in patients treated with dermal/fat transposition compared with no flap transposition. The authors believe that over-resection and excessive tissue tension, and possibly gluteal skin undermining and postoperative pressure, account for the increased risk.^22^ Patient satisfaction was not significantly improved by flap transposition.^22^

Autoaugmentation does not improve lateral gluteal deficiency, which is desired by many women to enhance an hourglass figure. Fat injection is simple and quick and provides a net increase in volume.^26^ It is imperative to stay subcutaneous when performing the injections to avoid fat emboli.^26^ The author has evaluated the level of fat injection using ultrasound in surgery to visualize the tissue planes; the cannula remains well above the muscle fascia.^27^

### Inner Thigh Lift

In recent years, a longitudinal excision of excess skin from the inner thighs^2,16,28–30^ has largely replaced the groin incision. A vertical vector is replaced with a horizontal vector.^29^ This operation leaves a scar along the inseam of the thigh, similar in concept to the brachioplasty scar. Vertical longitudinal skin resection is much more effective in correcting medial thigh skin laxity than the transverse groin approach.^17^ To avoid a T-junction in the groin crease, the author prefers a J-extension when necessary to chase the dog ear into the perineal crease.^31^ Treating the inner thigh with liposuction (analogous to the flank in an outer thigh lift) before undertaking the skin resection develops a safe dissection plane and reduces the volume of the extremity, allowing greater skin removal.^2^ The incision is not connected to the abdominoplasty incision (or scar) to avoid “framing” the pubic area.^4^

### Postoperative Care

Patients return to the office the day after surgery. Dressings are removed. A Doppler ultrasound examination is performed.^5^ It is possible to image the deep veins of the thigh even in patients undergoing inner thigh lifts. Patients start bathing the day after surgery. A standard elastic garment, either above or below the knee, is worn for 1 month. Exercising is typically resumed 1 month after surgery, but abdominal “core” exercises are deferred until at least 2 months after surgery.

No drain is used for an outer thigh/buttock lift. When the combined procedure is performed, a single drain is used, exiting through the right pubic portion of the abdominoplasty incision. The drain is removed in 3 or 4 days. By avoiding tissue undermining and using scalpel dissection exclusively,^7,32^ the risk of seromas is reduced.

### Complications

Complications are common after lower body lifts. Nemerofsky et al^1^ report a 50% complication rate with a dehiscence rate of 32.5%, and a skin necrosis rate of 9.5%. Ischemia from postoperative pressure on the sacrum and coccyx may contribute to impaired wound healing in the posterior midline.^1,33^ Seroma rates of about 20% are typical.^1,34^

Baca et al,^35^ in a study of 59 nonbariatric outpatients undergoing circumferential abdominoplasty, report that approximately half of their patients experienced a complication and 13.6% required a revision. Despite the frequency of complications, 90% of their patients stated that they would undergo the procedure again.^35^ Makipour et al^33^ report a 36% complication rate among patients undergoing outpatient circumferential lower body lifts. The most common complication was wound separation (24%), usually over the sacrum.^33^

Capella and Matarasso^2^ report that 45% of their massive weight loss patients experienced a complication after medial thigh lifts, primarily skin dehiscences (31%) occurring at the T intersection in the perineal crease and seromas (18%).

Buchanan et al^34^ treated 12 of their 19 lower body lift outpatients (63%) with antibiotics for cellulitis. Patients were initially treated prone and then repositioned supine. Baca et al^35^ report that 17.8% of their nonbariatric circumferential abdominoplasty patients were prescribed oral antibiotics for cellulitis. By contrast, Nemerofsky et al^1^ report infections in only 3.5% of their body lift patients, who are not repositioned prone to supine, similar to the findings in the present study, in which only 1 patient developed cellulitis (3%). Prepping the patients at the beginning of the case and avoiding patient repositioning from prone to supine avoid a potential break in sterility and may be effective in reducing infections.

Postoperative neuropathies can be minimized by careful attention to body positioning and padding during surgery.^17^ Avoiding deep dissection or sutures in the inguinal area reduces the risk of a lateral femoral cutaneous or iliohypogastric neuropathy.^8^

Persistent or recurrent skin laxity is common in massive weight loss patients and makes revisions inevitable in some patients.^17^ A revision rate of 26% was recently reported after outpatient circumferential lower body lifts.^33^ In the present study, 3 patients (8%) returned for secondary outer thigh lifts. Adequate subcutaneous fat resection at the posterior end of the resections reduces the need for dog ear revisions. Puckering of the tissue gradually settles down in most cases.

Doppler ultrasound screening is an effective method to detect deep venous thromboses and begin early treatment,^5^ as demonstrated by the affected patient in this series. Ultrasound is a safe alternative to chemoprophylaxis and avoids unnecessary bleeding and hematomas.^5^ Sequential compression devices do not reduce the risk of deep venous thromboses in plastic surgery outpatients treated with total intravenous anesthesia.^5^ Therefore, the author has discontinued their use.

Buchanan et al^34^ believe that avoiding hospitalization minimizes nosocomial infections and improves access to the surgery because of reduced cost. Inpatient surgery may represent a financial barrier for prospective patients.^33,36^

## CONCLUSIONS

Lower body lifts may be safely performed in the outpatient setting with attention to safe anesthesia, limited blood loss, and efficient use of operating room time. Preservation of a midline posterior skin bridge avoids elongation of the gluteal cleft. Superwet infusions assist with regional anesthesia and hemostasis and facilitate both liposuction and the dissections. Buttock fat injection is a safe adjunctive procedure to restore volume. Secondary surgery may be needed in some patients to treat persistent skin laxity.

## ACKNOWLEDGMENT

The author thanks Christina Engel, R.V.T., for data collection.
